# Turtle Flexion Reflex Motor Patterns Show Windup, Mediated Partly by L-type Calcium Channels

**DOI:** 10.3389/fncir.2017.00083

**Published:** 2017-10-31

**Authors:** Keith P. Johnson, Stephen M. Tran, Emily A. Siegrist, Krishna B. Paidimarri, Matthew S. Elson, Ari Berkowitz

**Affiliations:** ^1^Department of Biology, University of Oklahoma, Norman, OK, United States; ^2^Cellular and Behavioral Neurobiology Graduate Program, University of Oklahoma, Norman, OK, United States

**Keywords:** spinal cord, temporal summation, hindlimb, withdrawal, flexor, nifedipine

## Abstract

Windup is a form of multisecond temporal summation in which identical stimuli, delivered seconds apart, trigger increasingly strong neuronal responses. L-type Ca^2+^ channels have been shown to play an important role in the production of windup of spinal cord neuronal responses, initially in studies of turtle spinal cord and later in studies of mammalian spinal cord. L-type Ca^2+^ channels have also been shown to contribute to windup of limb withdrawal reflex (flexion reflex) in rats, but flexion reflex windup has not previously been described in turtles and its cellular mechanisms have not been studied. We studied windup of flexion reflex motor patterns, evoked with weak mechanical and electrical stimulation of the dorsal hindlimb foot skin and assessed via a hip flexor (HF) nerve recording, in spinal cord-transected and immobilized turtles *in vivo*. We found that an L-type Ca^2+^ channel antagonist, nifedipine, applied at concentrations of 50 μM or 100 μM to the hindlimb enlargement spinal cord, significantly reduced windup of flexion reflex motor patterns, while lower concentrations of nifedipine had no such effect. Nifedipine similarly reduced the amplitude of an individual flexion reflex motor pattern evoked by a stronger mechanical stimulus, in a dose-dependent manner, suggesting that L-type Ca^2+^ channels contribute to each flexion reflex as well as to multisecond summation of flexion reflex responses in turtles. We also found that we could elicit flexion reflex windup consistently using a 4-g von Frey filament, which is not usually considered a nociceptive stimulus. Thus, it may be that windup can be evoked by a wide range of tactile stimuli and that L-type calcium channels contribute to multisecond temporal summation of diverse tactile stimuli across vertebrates.

## Introduction

Windup is a form of temporal summation of somatosensory responses in which identical stimuli, delivered a second or more apart, rather than causing adaptation, trigger increasingly strong neuronal activity or behavioral responses (Herrero et al., 2000; Roca-Lapirot et al., 2017). Limb withdrawal reflex (flexion reflex) exhibits windup, at least in mammals (Herrero et al., 2000; Roca-Lapirot et al., 2017).

Cellular mechanisms of windup have been studied in a variety of *in vitro* preparations, as well as *in vivo*. Mechanisms of windup have been studied in slice preparations, especially in turtles (Russo and Hounsgaard, 1994, 1996; Russo et al., 1997) and rodents (Morisset and Nagy, 2000; Fossat et al., 2007; Roca-Lapirot et al., 2017). In both cases, both synaptic and intrinsic ion channels have been shown to contribute to windup. Evidence has accumulated that NMDA receptors normally play an important role in triggering the start of windup (Davies and Lodge, 1987; Dickenson and Sullivan, 1987; Fossat et al., 2007). In addition, postsynaptic L-type Ca^2+^ channels contribute importantly to continuing windup of dorsal horn neuronal responses to dorsal root stimulation in turtles (Russo and Hounsgaard, 1994, 1996; Russo et al., 1997) and to windup of both dorsal horn neuronal responses and flexion reflex responses to nociceptive stimuli in rats (Morisset and Nagy, 2000; Fossat et al., 2007; Roca-Lapirot et al., 2017). In subsets of dorsal horn and ventral horn interneurons, L-type Ca^2+^ channels mediate plateau potentials, the amplitude and duration of which increase with repeated stimulation; blocking L-type Ca^2+^ channels reduces or eliminates windup of these neurons’ responses (Russo and Hounsgaard, 1994, 1996; Russo et al., 1997; Svirskis and Hounsgaard, 1997). The main Ca^2+^ channel involved appears to be the Ca_V_1.3 channel (Perrier et al., 2002; Radwani et al., 2016). Opening of L-type calcium channels in turn can lead to opening of calcium-activated nonspecific cation channels, which can lead to afterdischarge (Morisset and Nagy, 1999; Aguiar et al., 2010; Roca-Lapirot et al., 2017).

While the influence of intrinsic properties such as plateau potentials has been reliably demonstrated in slice preparations, such intrinsic mechanisms cannot be assumed to have a large role during motor patterns in the intact spinal cord, because the large increase in conductance due to synaptic potentials may overwhelm the effects of intrinsic properties (Alaburda et al., 2005; Guzulaitis et al., 2016). Nonetheless, there is evidence that L-type Ca^2+^ channels contribute to plateau potentials in dorsal horn neurons during nociceptive flexion reflex in rats *in vivo* (Fossat et al., 2007; Reali et al., 2011; Radwani et al., 2016) and in an *ex vivo* turtle spinal cord-hindlimb preparation (Reali and Russo, 2013), as well as to motoneuron plateau potentials and the prolongation of leg flexion reflex in a frog spinal cord-hindlimb *ex vivo* preparation (Perrier and Tresch, 2005) and to nociceptive flexion reflex windup in rats *in vivo* (Fossat et al., 2007).

Key studies of the cellular mechanisms of windup have been conducted on turtle spinal cord slices (Russo and Hounsgaard, 1994, 1996; Russo et al., 1997) and in a turtle spinal cord-hindlimb preparation (Reali and Russo, 2013). These studies provided strong evidence that L-type calcium channels within spinal cord neurons contribute to windup of dorsal root-evoked and foot skin pinprick-evoked single-interneuron responses. Despite the key role of turtle spinal cord studies in the development of this field, turtle flexion reflex windup has not been described, nor its cellular mechanisms studied.

Ever since its discovery, windup has been shown to be due mainly to activation of sensory neurons with unmyelinated, slow-conducting axons, C-fibers, most of which are nociceptive (Mendell and Wall, 1965; Mendell, 1966; Price, 1972; Magerl et al., 1998; Herrero et al., 2000). Thus, windup has generally been associated with nociceptive processing. It is not clear whether innocuous skin stimuli can also trigger windup. Recently, it was shown that weak electrical stimulation of hindlimb nerves can trigger flexion reflex windup in cats (Frigon et al., 2012; Johnson et al., 2017). It is not known, however, whether innocuous tactile stimulation of the foot skin can also trigger windup.

We show here that mechanical and electrical stimulation of the dorsal foot skin triggers windup of flexion reflex motor patterns in turtles *in vivo*. We find that flexion reflex windup can be evoked even using very weak mechanical stimulation that is likely innocuous. In addition, using the dihydropyridine L-type Ca^2+^ channel antagonist nifedipine, we show that L-type Ca^2+^ channels contribute to this turtle flexion reflex windup, as was shown previously for other kinds of windup. Our findings suggest that similar multisecond temporal summation mechanisms involving L-type Ca^2+^ channels contribute to normal sensory processing of a wide range of tactile stimuli across vertebrates.

## Materials and Methods

### Animal Preparations

Adult red-eared turtles (*Trachemys scripta elegans*, both sexes, *n* = 32), were placed in crushed ice for 2 h before surgery to induce hypothermic analgesia and were kept in ice throughout all surgical procedures. Surgical procedures have been previously described (Robertson et al., 1985) and are briefly outlined here. The spinal cord was first exposed and transected between the dorsal (D) 2 and D3 dorsal roots. Softened dental wax was used to cover this exposure site. The five segments of the spinal cord hindlimb enlargement (D8–D10 and sacral (S) 1–S2) and two pre-enlargement segments (D6–D7) were then exposed. All layers of the meninges were torn over the length of this exposure to allow drugs to diffuse into the spinal cord. This spinal cord exposure was encircled by a wax well molded onto the carapace. The right hip flexor (HF) muscle nerve, ventral puboischiofemoralis internus, pars anteroventralis, was dissected free for recording. After surgery, turtles were removed from the ice, warmed to room temperature, immobilized with gallamine triethiodide (8 mg/kg i.m.; Sigma-Aldrich, St. Louis, MO, USA), and then artificially ventilated (Harvard Apparatus, Holliston, MA, USA) throughout the experiment. All animal procedures were approved by the Institutional Animal Care and Use Committee of the University of Oklahoma.

### Nerve Recordings

Recordings began 12–18 h after completion of the surgical preparation. The dissected nerve was submerged in mineral oil and surrounded by a wax well molded onto the carapace. Recordings from the nerve were obtained extracellularly with a pair of 100-μm silver wires. Filtered and amplified (×1000; band pass 0.1–1.0 kHz; A-M Systems, Carlsborg, WA, USA and Natus Neurology-Grass, Warwick, RI, USA) nerve activity was recorded on a digital audio tape recorder (TEAC America, Montebello, CA, USA).

### Stimulation

Each individual flexion reflex was evoked by stimulation of the right hindlimb dorsal foot skin, using either a brief tap with a von Frey filament (2–10 g; Touch Test, North Coast Medical, Gilroy, CA, USA) or a train of five 1-ms pulses (4–76 V) at 100 Hz via a pair of cup electrodes attached with electrode cream, with their centers about 1 cm apart (Astromed-Grass electrodes, Warwick, RI, USA; Master-8 stimulator with ISO-Flex stimulus isolation unit, A.M.P.I., Jerusalem, Israel). A series of 3–5 such mechanical or electrical stimuli (to a single location) with 3–5 s interstimulus intervals was used to trigger flexion reflex windup. For mechanically evoked flexion reflex windup, we used a metronome app to time interstimulus intervals. For electrically evoked flexion reflex windup, we adjusted the stimulus voltage such that the first stimulus in the series evoked a just detectable flexion reflex response. Only three stimuli were delivered systematically and only the first three responses were studied quantitatively because rhythmic motor patterns (probably scratching, but we did not investigate this further) often began in the HF nerve during later stimuli and these would have confounded analysis of later flexion reflex responses. In most experiments, windup trials were alternated with trials in which a single, stronger mechanical stimulus (usually using a 10-g von Frey filament) was used to evoke a flexion reflex, so that we could assess the effect of nifedipine on a single flexion reflex and assess the hypothesis that summation of late components of flexion reflex contributes to flexion reflex windup. A 3-min rest was given between each trial and the next. In each animal used to test the effects of nifedipine, the interstimulus interval and number of stimuli were chosen to maximize windup, then kept constant throughout the experiment. In nifedipine and Bay K 8644 experiments, after several pre-drug trials, control saline was replaced with the drug solution over the spinal cord and trials were continued with no additional break. To verify that the quality of the HF recording was unchanged during the experiment, we evoked rostral scratching via mechanical stimulation of the shell rostral to the region innervated by the D6-S2 segments, before and during drug application (Stein and Schild, 1989).

### Pharmacology

Nifedipine (Sigma-Aldrich, St. Louis, MO, USA) was dissolved initially in 200 μL dimethylsulfoxide (DMSO; Sigma-Aldrich, St. Louis, MO, USA) and then added to turtle saline (133 mM NaCl, 3.8 mM KCl, 2.8 mM CaCl_2_, 2.2 mM MgCl_2_, 5 mM Trizma pH 7.4 crystals) to a final concentration of 3 μM, 12 μM, 25 μM, 50 μM or 100 μM. Bay K 8644 (Sigma-Aldrich, St. Louis, MO, USA) was dissolved initially in 200 μL DMSO and then added to turtle saline to a final concentration of 2 μM, 10 μM, 20 μM, 50 μM, 100 μM, or 200 μM. Either nifedipine solution or Bay K8644 solution was added to the D6-S2 spinal cord exposure following control trials and removal of control saline (which did not contain DMSO); in nifedipine experiments, the wax well was then covered with aluminum foil to protect the spinal cord from exposure to light. In some animals, multiple concentrations of nifedipine were tested; in these cases, solutions were tested in order of increasing concentration because we found that nifedipine could not be completely washed out of the intact spinal cord.

### Data Analysis

**Figure 1:**
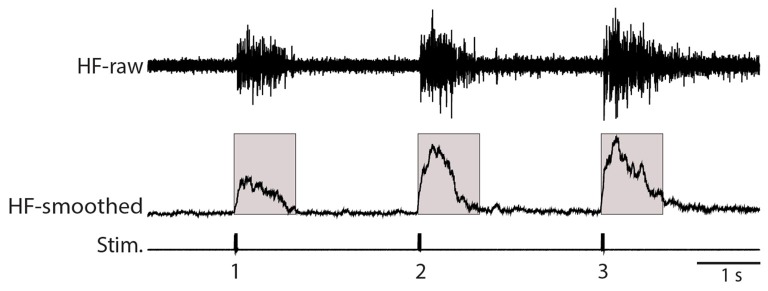
Illustration of analysis methods. The raw recording of the hip flexor (HF) nerve (top, HF-raw) was rectified and smoothed using a 100-ms sliding window (middle, HF-smoothed). The mean value of this rectified and smoothed signal (with the baseline adjusted to zero) was calculated over the 1.0 s following each stimulus (indicated by the shaded boxes). Stim., stimuli.

Nerve recordings were digitized at 5 kHz, rectified, and smoothed (100-ms sliding window), using Datapac (Run Technologies, Laguna Hills, CA, USA). We measured the mean amplitude of each HF nerve response during the 1-s epoch following each stimulus (see Figure 1), after adjusting the (pre-stimulus) baseline voltage to zero. Flexion reflex responses to a single strong mechanical stimulus were normalized to the mean pre-drug response to the same stimulus. For quantitative analysis of windup, responses were normalized to the mean response to the 3rd stimulus in that condition so that windup could be quantified independent of the absolute magnitude of the HF nerve response. The windup index was calculated as (response 3 − response 1)/(mean 3rd response pre-drug − mean 1st response pre-drug), such that its value would have a mean of 1.0 for pre-drug trials, with >1.0 indicating that the drug increased windup and <1.0 indicating that the drug decreased windup. In figures, these values were multiplied by 100 to yield percentages of pre-drug values. To compare statistically the mean responses to any two consecutive stimuli (1st vs. 2nd or 2nd vs. 3rd), we used the two-tailed Mann-Whitney test, with significance set at *p* < 0.05. To compare statistically the amplitude of an individual flexion reflex in control saline vs. nifedipine and to compare the windup index value in control saline vs. drug application, we used the one-tailed Mann-Whitney test, with significance set at *p* < 0.05, because we hypothesized that L-type Ca^2+^ channels contribute to flexion reflex and to flexion reflex windup. To compare flexion reflex responses to multiple nifedipine concentrations within an animal, we used analysis of variance (ANOVA). Statistical tests were performed using InStat (GraphPad Software, La Jolla, CA, USA).

## Results

### Flexion Reflex Windup

**Figure 2:**
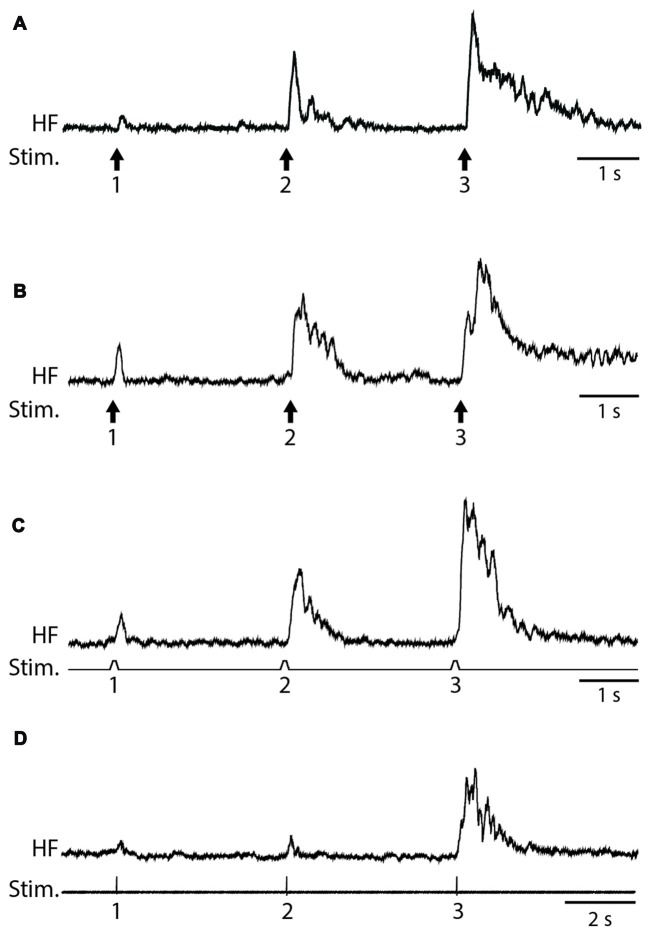
Examples of flexion reflex windup. **(A)** 2-g (20-mN) von Frey filament taps to the dorsal hindlimb foot skin, with 3-s interstimulus intervals. **(B)** 4-g (39-mN) von Frey filament taps to the dorsal hindlimb foot skin, with 3-s interstimulus intervals. **(C)** Electrical pulses to the dorsal hindlimb foot skin, with 3-s interstimulus intervals. **(D)** Electrical pulses to the dorsal hindlimb foot skin, with 5-s interstimulus intervals. HF, hip flexor nerve recording (rectified and smoothed); Stim., stimuli.

We used weak von Frey filament stimuli in some experiments to best mimic natural stimulation and used electrical skin stimuli in other experiments to maximize the consistency of stimulus amplitude, duration and timing. The amplitude of flexion reflex motor patterns (henceforth, just “flexion reflex”), as assessed via the mean HF amplitude over 1 s following the stimulus (Figure 1), consistently increased within a series of weak mechanical (2-g (20 mN; Figure 2A) or 4-g (39-mN; Figure 2B) von Frey filament) or electrical (Figure 2C) stimuli to the dorsal hindlimb skin that were 3 s apart. We could evoke flexion reflex windup using a 4-g von Frey filament in all animals tested (*n* = 5) and using a 2-g von Frey filament in an additional animal (Figure 2A). Using electrical stimuli, we found that clear flexion reflex windup could be evoked with interstimulus intervals as long as 5 s (Figure 2D), though typically not as strongly as with 3-s intervals. Flexion reflex windup was not seen with 10-s interstimulus intervals (data not shown). The interstimulus intervals with which we evoked flexion reflex windup were similar to or greater than those used in previous studies of spinal interneuron windup, nociceptive flexion reflex windup and scratch-evoking tactile stimulus temporal summation (Currie and Stein, 1988, 1990; Russo and Hounsgaard, 1994, 1996; Russo et al., 1997; Morisset and Nagy, 2000; Fossat et al., 2007; Reali et al., 2011; Frigon et al., 2012; Reali and Russo, 2013).

### Effect of Nifedipine on an Individual Flexion Reflex

**Figure 3:**
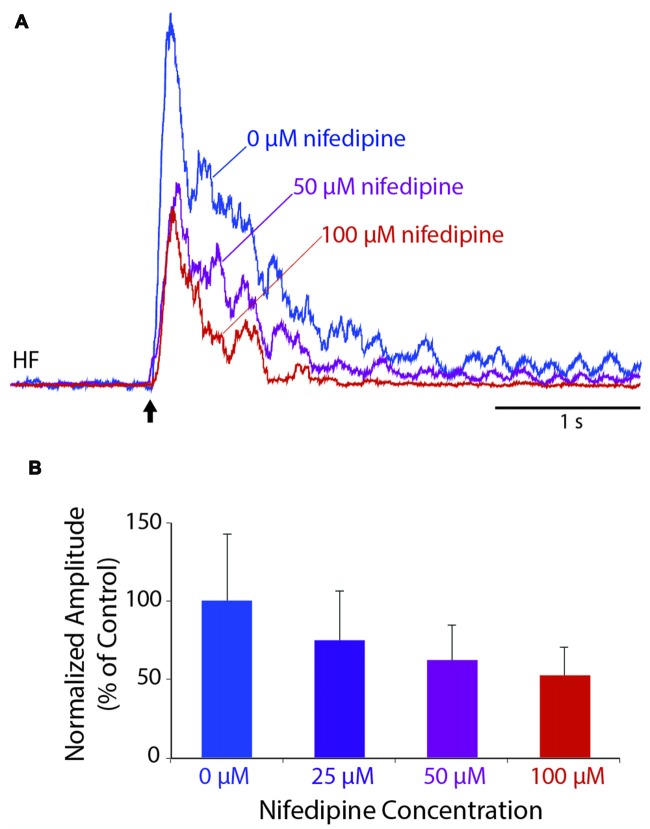
Dose-dependent reduction of an individual flexion reflex by the L-type Ca^2+^ channel blocker, nifedipine. **(A)** Example traces from the HF nerve in response to a 10-g von Frey filament tap to the dorsal hindlimb foot skin, before and during nifedipine application. **(B)** Dose-dependent reduction in mean amplitude of the HF burst (during the 1-s epoch following each stimulus, normalized to the mean amplitude in control saline) for the animal shown in **(A)**. Error bars are standard deviations. Flexion reflex was significantly reduced by 100 μM nifedipine compared to control saline (see “Results” section). All trials were included beginning 15 min after drug application and ending 60 min later or whenever the nifedipine solution was replaced with a higher concentration, whichever came first.

To test whether L-type Ca^2+^ channels contribute to an individual flexion reflex, we applied a single tap to the hindlimb dorsal skin with a 10-g von Frey filament to evoke a strong flexion reflex, before and after applying solutions of nifedipine, a dihydropyridine antagonist of L-type Ca^2+^ channels, to the D6-S2 spinal cord. We found that nifedipine reduced the amplitude of flexion reflex in a dose-dependent manner, shown in Figure 3 for a representative animal (ANOVA: *p* = 0.025; control (*n* = 8 trials), 50 μM (*n* = 6), and 100 μM (*n* = 6) were compared statistically, but 25 μM was not included, because the n (4) was too small to assess normality). Flexion reflex mean amplitude was significantly reduced by 100 μM nifedipine compared to control saline in five of seven animals tested (*p* < 0.05; Mann-Whitney test). Lower concentrations of nifedipine (50 μM, *n* = 2; 25 μM, *n* = 2; 3 μM, *n* = 3) had no statistically significant effect on the mean amplitude of an individual flexion reflex.

### Effect of Nifedipine on Flexion Reflex Windup

**Figure 4:**
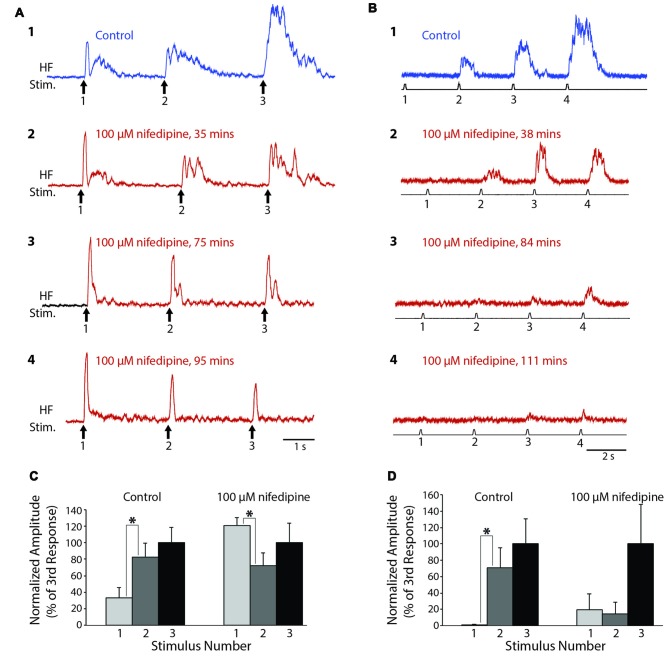
Progressive reduction in flexion reflex windup during nifedipine application to the spinal cord. **(A)** Examples of windup elicited by 4-g von Frey filament taps to the dorsal hindlimb foot skin with approximately 3-s intervals in control saline **(1)** and during 100 μM nifedipine application to the spinal cord **(2–4)**. **(B)** Examples of windup elicited by electrical stimulation of the dorsal hindlimb foot skin at 3-s intervals in control saline **(1)** and during 100 μM nifedipine application to the spinal cord **(2–4)**. **(C,D)** Mean flexion reflex amplitude (normalized to the mean response to the 3rd stimulus in the same condition) in control saline and 100 μM nifedipine for the animals shown in **(A,B)** respectively. Error bars are standard deviations. Asterisks indicate statistically significant increases in mean response amplitude between consecutive stimuli; other differences in mean responses between consecutive stimuli were not statistically significant. In **(C)** there were 12 control trials and 10 nifedipine trials; in **(D)** there were five control trials and 11 nifedipine trials.

To test whether L-type Ca^2+^ channels also contribute to turtle flexion reflex windup, we delivered a series of weak mechanical or electrical stimuli to the dorsal hindlimb foot skin before and after applying nifedipine to the D6-S2 spinal cord. In control saline, clear flexion reflex windup was evoked using a 4-g von Frey filament and 3-s interstimulus intervals (Figure 4A1). Following the application of 100 μM nifedipine, this mechanically evoked flexion reflex windup was gradually reduced (Figures 4A2–4). In this animal, the initial component of flexion reflex remained even in the presence of nifedipine, but windup of flexion reflex was completely eliminated. In control saline, the mean response in this animal increased from the 1st to the 2nd stimulus and from the 2nd to the 3rd stimulus; the increase from the 1st to the 2nd stimulus was statistically significant (Figure 4C; *p* < 0.05; Mann-Whitney test). In nifedipine, however, these increases disappeared and the mean response actually decreased significantly from the 1st to the 2nd stimulus (Figure 4C). The mean value of the windup index (i.e., the change in response from the 1st to the 3rd stimulus, normalized to the mean pre-drug change; see “Materials and Methods” section) decreased from a mean ± SD of 1.0 (by definition) ± 1.44 in control saline to −0.32 ± 1.27 in nifedipine.

In a different animal, we used a series of weak electrical stimuli to the dorsal hindlimb skin to evoke flexion reflex windup (Figure 4B1). Similar to the mechanically evoked windup, the electrically evoked windup was gradually reduced by 100 μM nifedipine (Figures 4B2–4). The electrically evoked flexion reflex responses were almost completely eliminated by nifedipine in this animal, though some windup of the remaining weak responses continued. In this case, the mean response increased significantly from the 1st to the 2nd stimulus in control saline but not in nifedipine. Also, the mean ± SD of the windup index decreased from 1.0 ± 0.62 in control saline to 0.012 ± 0.29 in nifedipine (Note that there was still an apparent, though not statistically significant, increase in the mean response from the 1st to the 3rd stimulus in nifedipine (Figure 4D, right), but the absolute amount of this increase was much smaller than in control saline, so the windup index value in nifedipine was very low).

**Figure 5:**
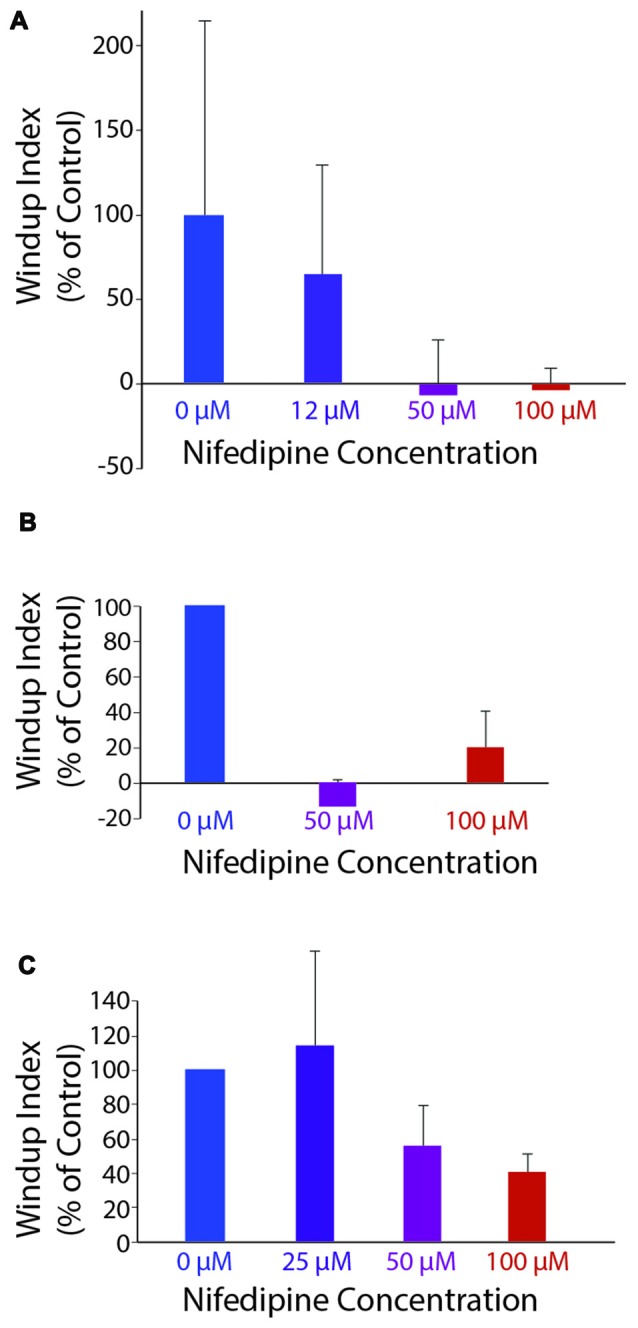
Windup index (set at 100% in control saline; see “Materials and Methods” section) as a function of nifedipine concentration. **(A)** Mechanically evoked (4-g von Frey filament) flexion reflex windup, in one animal. **(B)** Mechanically evoked flexion reflex windup across all animals for concentrations of nifedipine tested in more than one animal (*n* = 5, 2 and five animals for control, 50 μM and 100 μM, respectively). **(C)** Electrically evoked flexion reflex windup across all animals for concentrations of nifedipine tested in more than one animal (*n* = 13, 3, 4 and 13 animals for control, 25 μM, 50 μM, and 100 μM, respectively). Error bars are standard deviation for **(A)** and standard error of the mean for **(B,C)**.

Mechanically evoked flexion reflex windup was significantly reduced by nifedipine in a dose-dependent manner, as assessed by reduction in the mean value of the windup index, shown for a representative animal in Figure 5A (ANOVA, *p* = 0.04). Mean windup index values for mechanically evoked flexion reflex with multiple concentrations of nifedipine are shown across animals in Figure 5B. Mechanically evoked flexion reflex windup index was significantly reduced (*p* < 0.05; Mann-Whitney test) by 100 μM nifedipine in three of five animals tested, by 50 μM nifedipine in two of two animals tested, by 25 μM nifedipine in zero of one animal tested, and by 12 μM nifedipine in zero of one animal tested.

Electrically evoked flexion reflex windup was also reduced by nifedipine in a dose-dependent manner, as assessed by reduction in the mean value of the windup index, shown across animals in Figure 5C. Electrically evoked flexion reflex windup index was significantly reduced (*p* < 0.05; Mann-Whitney test) by 100 μM nifedipine in 7 of 13 animals tested, by 50 μM nifedipine in two of four animals tested, by 25 μM nifedipine in one of three animals tested, by 12 μM nifedipine in zero of one animal tested, and by 3 μM nifedipine in zero of one animal tested. The variability of flexion reflex responses may account for the effect of 100 μM nifedipine not being statistically significant in some animals.

In each of four animals, we were able to test the effect of one or more concentrations of nifedipine on both mechanically evoked and electrically evoked flexion reflex windup. In these animals, the mean windup index was 0.26 ± 0.25 (mean ± SEM) for mechanically evoked windup and 0.29 ± 0.16 for electrically evoked windup in 100 μM nifedipine (*n* = 4), −0.13 ± 0.005 and 0.31 ± 0.17, respectively, in 50 μM nifedipine (*n* = 2), 1.003 and 2.25, respectively, in 25 μM nifedipine (*n* = 1), and 0.75 and 1.34, respectively, in 12 μM nifedipine (*n* = 1).

In two of the four animals, the reduction in windup by 100 μM nifedipine was statistically significant (Mann-Whitney test) for mechanically evoked (*p* = 0.018 and 0.006) and electrically evoked (*p* = 0.003 and 0.038) windup. In another animal, the reduction in windup by 100 μM nifedipine was statistically significant for electrically evoked (*p* = 0.007) but not mechanically evoked (*p* = 0.063) windup. In the remaining animal, nifedipine significantly reduced neither kind of windup. Fifty micromolar nifedipine significantly reduced both mechanically evoked (*p* = 0.002) and electrically evoked (*p* = 0.013) windup in 1 animal; it significantly reduced mechanically evoked (*p* = 0.044) but not electrically evoked (*p* = 0.37) windup in another animal. Neither 25 μM nor 12 μM significantly reduced either mechanically evoked or electrically evoked windup (*n* = 1 animal each).

Thus, in the animals for which direct comparisons were possible, 100 μM nifedipine was effective in reducing windup using either mechanical or electrical stimulation, while 12–25 μM nifedipine was ineffective in reducing windup using either mechanical or electrical stimulation. The fact that 25 μM and lower concentrations of nifedipine (initially dissolved in the same amount of DMSO as higher concentrations of nifedipine were) had no effect on either mechanically or electrically evoked flexion reflex windup also demonstrates that the DMSO itself had no measurable effect on flexion reflex windup.

### Effect of Bay K 8644 on Flexion Reflex Windup

**Figure 6:**
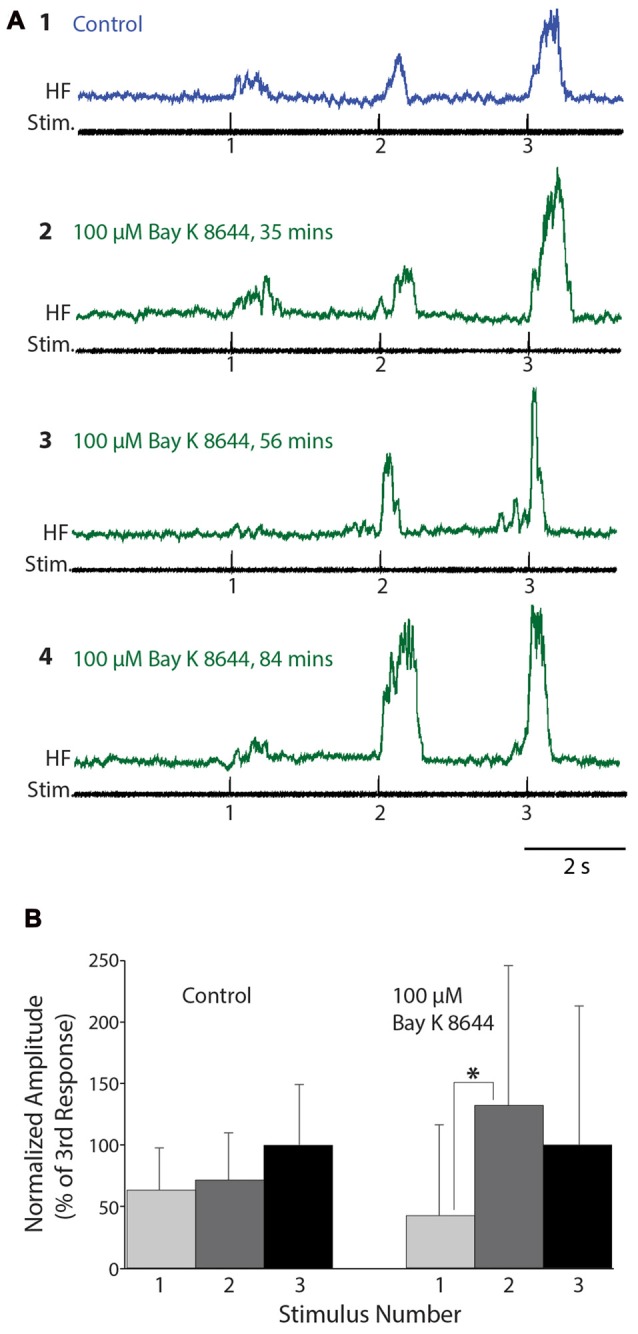
Flexion reflex windup before and during 100 μM Bay K 8644 application to the spinal cord. **(A)** Example flexion reflex responses in control saline **(1)** and 100 μM Bay K 8644 **(2–4)** using electrically evoked flexion reflex and 3-s intervals. **(B)** Mean flexion reflex amplitude (normalized to the mean response to the 3rd stimulus) in control saline and during 100 μM Bay K 8644 application for the animal shown in **(A)**. Error bars are standard deviations. Asterisk indicates a statistically significant increase in mean response amplitude from the 1st to the 2nd stimulus in Bay K 8644; other differences between consecutive mean responses were not statistically significant. There were 14 control trials and 16 Bay K 8644 trials.

We also tested the effects of Bay K 8644, which enhances the activity of L-type Ca^2+^ channels, on flexion reflex windup evoked by mechanical and electrical stimulation of the hindlimb foot skin (*n* = 16 animals). In concentrations of 50 μM or less (2 μM: *n* = 2; 10 μM: *n* = 4; 20 μM: *n* = 2; 50 μM: *n* = 2), no clear effects on flexion reflex windup were seen, though spontaneous activity in the HF nerve typically increased (not shown). We also tested higher concentrations of Bay K 8644 (100 μM: *n* = 5; 200 μM: *n* =1). In some experiments using 100 μM Bay K 8644, however, flexion reflex windup did appear to increase; examples are shown for one animal in Figure 6. In this case, the mean flexion reflex response was significantly larger for the second stimulus than the first stimulus in Bay K 8644, but not in control saline. The mean ± SD of the windup index increased from 1.0 ± 1.53 in control saline to 1.58 ± 3.65 in Bay K 8644; nonetheless, this windup index increase was not statistically significant due to the large standard deviation with Bay K 8644. Variability of responses in Bay K 8644 (and thus standard deviation) was also large in the four other animals tested with 100 μM and the one animal tested with 200 μM Bay K 8644, which also did not show a statistically significant increase in the windup index.

## Discussion

### L-Type Calcium Channels Contribute to Turtle Flexion Reflex

We found that nifedipine, a dihydropyridine L-type calcium channel antagonist, significantly reduced the amplitude of turtle flexion reflex motor patterns *in vivo*. NMDA receptor antagonists have previously been shown to reduce the amplitude of turtle flexion reflex motor patterns (Stein and Schild, 1989), suggesting that both NMDA receptors and L-type Ca^2+^ channels contribute to turtle flexion reflex. L-type Ca^2+^ channels have previously been shown to contribute to flexion reflex in frogs (Perrier and Tresch, 2005) and mammals (Fossat et al., 2007). Perrier and Tresch (2005) suggested that the persistent inward current produced by L-type Ca^2+^ channels serves to increase the duration of motoneuron depolarization and thus prolong the flexion reflex. It is likely that the opening of NMDA receptor channels contributes to depolarizing neurons sufficiently to trigger the opening of L-type calcium channels (Fossat et al., 2007).

### Turtle Flexion Reflex Shows Windup, Mediated Partly by L-Type Calcium Channels

We report here for the first time that turtle flexion reflex shows windup and that L-type calcium channels contribute to this windup. Mechanically and electrically evoked flexion reflex windup *in vivo* were both significantly and consistently reduced when an L-type Ca^2+^ channel antagonist, nifedipine, was applied to the spinal cord. Also, Bay K 8644, which enhances the effects of L-type Ca^2+^ channels, could increase flexion reflex windup, though it also increased variability of responses, perhaps because it enhanced Ca^2+^ currents that play unrelated roles in addition to those that mediate flexion reflex windup. Nifedipine and Bay K 8644 affect multiple subtypes of L-type calcium channels, of which mainly Ca_v_1.2 and Ca_v_1.3 occur in the spinal cord (Roca-Lapirot et al., 2017). These channels can mediate plateau potentials and windup, as well as triggering afterdischarge via calcium-dependent nonspecific cation channels. These cellular effects can contribute to multiple forms of normal plasticity, as well as a variety of neurological disorders (Roca-Lapirot et al., 2017).

The current findings add to previous evidence, initially from turtle spinal cord slice studies, that L-type calcium channels contribute importantly to dorsal root-evoked windup of deep dorsal horn plateau-generating neurons’ responses and to windup or “warmup” of both deep dorsal horn and ventral horn interneuron responses to intracellular depolarization (Russo and Hounsgaard, 1994, 1996; Russo et al., 1997; Svirskis and Hounsgaard, 1997), as well as to windup of deep dorsal horn plateau-generating neurons’ responses to foot skin pinprick in a turtle spinal cord-hindlimb integrated preparation (Reali and Russo, 2013). L-type calcium channels have also been shown to contribute importantly to windup of deep dorsal horn plateau-generating neurons’ responses to dorsal root stimulation and intracellular depolarization in rat spinal cord slices (Morisset and Nagy, 1996, 2000) and to flexion reflex windup in rats *in vivo* (Fossat et al., 2007). Collectively, these studies suggest that L-type calcium channels contribute importantly to multiple kinds of windup in both mammalian and non-mammalian vertebrates.

The long-lasting components of consecutive flexion reflexes might in principle summate to generate windup. However, nifedipine-sensitive windup of turtle spinal cord neurons’ responses can occur even when the membrane potential returns to baseline prior to the next stimulus (Perrier et al., 2002), suggesting that depolarization-evoked facilitation of L-type Ca^2+^channels can mediate windup even without cumulative depolarization between stimuli. Thus, both L-type Ca^2+^ channel-mediated cumulative depolarization and L-type Ca^2+^ channel facilitation may contribute to flexion reflex windup.

### Flexion Reflex Windup Can be Triggered by Weak Tactile Stimulation

We were consistently able to evoke flexion reflex windup by stimulating the dorsal foot skin with a 4-g (39-mN) von Frey filament and in some cases with a 2-g (20-mN) von Frey filament. In rats, even a 10-g von Frey filament stimulation is regarded as innocuous (Reali et al., 2011). The threshold for evoking a hindlimb flexion reflex in rats using a von Frey filament has been reported to be 1.1–3.6 g (Chaplan et al., 1994) and 5.1 g (Rigaud et al., 2011), though, of course, a flexion reflex does not necessarily indicate pain. Use of von Frey filament stimulation and flexion reflex to assess pain in animals is necessarily problematic (Wu et al., 2010), but using behavioral criteria, pain threshold in rats with von Frey filament stimulation of the hindpaw was found to be about 60 g (Reali et al., 2011; M’Dahoma et al., 2014). The average force threshold for von Frey filament skin stimulation to be perceived as painful by human subjects has been reported to be 5 g (49 mN; Ziegler et al., 1999) and 84.6 g (830 mN; Magerl et al., 1998). Thus, the mechanically evoked flexion reflex windup we report here was obtained with a von Frey filament force level that is likely to be innocuous, given previous findings across vertebrates. It was previously shown that weak electrical stimulation of hindlimb nerves could trigger flexion reflex windup in cats (Frigon et al., 2012; Johnson et al., 2017), but this is the first demonstration, to our knowledge, that tactile skin stimuli likely to be innocuous can also trigger flexion reflex windup. Although nociceptive flexion reflex is commonly used to assess altered nociceptive processing, it has long been noted that innocuous tactile stimuli can also evoke flexion reflex (Sherrington, 1910; Sandrini et al., 2005; Duysens et al., 2013). Our results suggest that windup may occur in response to a wide range of tactile stimuli, not just nociceptive stimuli, and thus may be a component of tactile sensory processing generally. Our finding that nifedipine reduced flexion reflex windup evoked by weak mechanical stimulation raises the possibility that L-type calcium channels contribute to multisecond temporal summation mechanisms for both nociceptive and innocuous tactile stimuli.

A possible explanation for our ability to evoke flexion reflex windup using weak stimuli, despite previous suggestions that flexion reflex windup requires nociceptive stimuli, is that the spinal transection in our experiments produced allodynia. While we cannot rule this out conclusively, we think that it is unlikely given the brief delay (typically 12–18 h) between spinal transection and flexion reflex testing (see “Materials and Methods” section). M’Dahoma et al. (2014), for example, did not observe a change in pain threshold to von Frey filament stimulation of the hindpaw in spinal cord-transected rats for at least 9 days following transection. Schouenborg et al. (1992) did see reduced flexion reflex thresholds several hours after spinalization in rats, but the threshold only went down to about 100 mN and this was using a “pincher”, not a von Frey filament.

From its earliest descriptions, windup has been associated with the activation of sensory neurons with unmyelinated, slow-conducting axons, C-fibers, most of which are nociceptive, both in humans and in non-human animals (Mendell and Wall, 1965; Mendell, 1966; Price, 1972; Magerl et al., 1998; Herrero et al., 2000). Although many C-fibers are nociceptive, other C-fibers respond instead to gentle skin stimulation in cats (Zotterman, 1939; Douglas and Ritchie, 1957; Iggo, 1960; Bessou and Perl, 1969; Bessou et al., 1971), mice (Li et al., 2011), rats (Leem et al., 1993), guinea pigs (Lawson et al., 1997), rabbits (Shea and Perl, 1985), monkeys (Kumazawa and Perl, 1977), and humans (Nordin, 1990; Vallbo et al., 1993, 1999). In mammals, these sensory neurons mostly (Torebjörk, 1974; Olausson et al., 2010; Abraira and Ginty, 2013) but not exclusively (Djouhri, 2016) innervate hairy skin. Thus, the flexion reflex windup shown here might be mediated by C-fibers, despite the stimulation likely being innocuous. Low-threshold mechanoreceptors have also been shown to contribute to paw withdrawal in normal mice (Arcourt et al., 2017). Finally, it is possible that the flexion reflex windup found here in response to weak mechanical stimulation is mediated by A∂ fibers, consistent with some previous findings in mice (Kimura and Kontani, 2008).

### Other Kinds of Multisecond Excitability Changes in Turtle Spinal Cord

Multisecond temporal summation has also been demonstrated using weak electrical stimulation of the turtle shell to evoke scratching motor patterns (Currie and Stein, 1988, 1990). Currie and Stein (1992) identified a subset of interneurons that produce prolonged afterdischarges following brief peripheral stimulation that triggers scratching. They found that NMDA receptors contribute to the long afterdischarge of these sensory interneurons as well as to the multisecond excitability of scratching motor patterns, but did not test the contribution of L-type Ca^2+^ channels to these multisecond excitability changes. Just after a scratching episode, however, turtle motoneurons have been shown to have increased excitability that is sensitive to nifedipine and to a metabotropic glutamate antagonist (Alaburda and Hounsgaard, 2003), suggesting that facilitation of L-type Ca^2+^ channels contributes to elevated neuronal excitability for a few seconds following each scratching episode. Despite the demonstrated contribution of L-type Ca^2+^ channels to motoneuron excitability following scratching, neuronal intrinsic properties generally may have little effect during a scratching or swimming motor pattern itself, due to the overwhelming conductance increase produced by synaptic potentials during these motor patterns (Alaburda et al., 2005; Guzulaitis et al., 2016). Yet, L-type Ca^2+^ channels do make a substantial contribution both within a flexion reflex in turtles (shown here) and frogs (Perrier and Tresch, 2005) and to flexion reflex windup in turtles (shown here) and rats (Fossat et al., 2007), as well as to windup of deep dorsal horn neuron responses to foot pinprick stimulation in turtles (Reali and Russo, 2013). Thus, L-type Ca^2+^ channels may generally play a larger role within a flexion reflex and in windup of both flexion reflex and scratching motor patterns than they play within an episode of a rhythmic motor pattern.

## Author Contributions

EAS, MSE and AB conceived and designed the research. KPJ, SMT, EAS, KBP and MSE performed the experiments. KPJ, SMT, EAS, KBP and AB analyzed the data. KPJ, SMT, EAS, KBP and AB interpreted the results of the experiments. KPJ drafted the manuscript. AB edited and revised the manuscript. All authors have approved the final version of the manuscript and agree to be accountable for all aspects of the work. All persons designated as authors qualify for authorship, and all those who qualify for authorship are listed.

## Conflict of Interest Statement

The authors declare that the research was conducted in the absence of any commercial or financial relationships that could be construed as a potential conflict of interest.
